# Drivers of Wolf Activity in a Human‐Dominated Landscape and Its Individual Variability Toward Anthropogenic Disturbance

**DOI:** 10.1002/ece3.70397

**Published:** 2024-10-22

**Authors:** Iago Ferreiro‐Arias, Emilio José García, Vicente Palacios, Víctor Sazatornil, Alejandro Rodríguez, José Vicente López‐Bao, Luis Llaneza

**Affiliations:** ^1^ Department of Conservation Biology and Global Change Estación Biológica de Doñana, CSIC Sevilla Spain; ^2^ ARCA, People and Nature Oviedo Spain; ^3^ Conservation Biology Group Forest Science and Technology Centre of Catalonia (CTFC) Solsona Spain; ^4^ Biodiversity Research Institute (University of Oviedo—CSIC—Principado de Asturias) Oviedo University Mieres Spain; ^5^ A.RE.NA.—Asesores en Recursos Naturales Lugo Spain; ^6^ Facultade de Ciencias, Área de Zooloxía, Grupo de Investigación en Biología Evolutiva (GIBE) Universidade da Coruña A Coruña Spain

**Keywords:** behavioral variability, boldness, *Canis lupus*, individual response, refuge cover, temporal avoidance

## Abstract

Wolves (*Canis lupus)* exhibit contrasted activity patterns along their distribution range. The shift from diurnal to nocturnal habits within and among populations appears to be primarily driven by localized levels of human activity, with ambivalent responses toward such disturbance reported among populations. Yet, the drivers and the underlying individual variability of temporal avoidance patterns toward human remains unexplored. We equipped 26 wolves with GPS–GSM collars, obtaining 54,721 locations. We used step lengths, turning angles, and accelerometer data from recorded locations to infer activity through hidden Markov models (Conners, M. G., T. Michelot, E. I. Heywood, et al. 2021. “Hidden Markov Models Identify Major Movement Modes in Accelerometer and Magnetometer Data From Four Albatross Species.” *Movement Ecology* 9, no. 1: 1–16.). We further explored the probability of activity as a function of a set of proxies of anthropogenic disturbance at different spatial scales and its interaction with different periods of the day by fitting population‐level and individual‐based hidden Markov models. Wolves were predominantly active during dusk and night, yet variations in activity emerged among individuals across day periods. We did not find clear population‐level effects of anthropogenic disturbance predictors, as these were masked by a wide range of individual‐specific responses, which varied from positive to negative, with inter‐individual variability in responses changing according to different predictors and periods of the day. Our results suggest a non‐uniform strategy of wolves in adapting their behavior to human‐dominated environments, further underscoring the role of vegetation patches acting as functional refuge cover for buffering the effects of anthropogenic disturbance and boosting the persistence of the species in human‐dominated landscapes. This study, for the first time, reveals the individual variability in wolf responses to human disturbance. By fitting hidden Markov models to data from GPS–GSM collars deployed on 26 wolves, we found significant variation between individuals in their responses to different levels of anthropogenic pressure and across different times of day, highlighting a non‐uniform strategy for coping with perturbations in human‐dominated landscapes. Our findings underscore the diverse behavioral adjustments employed by wolves to persist in these environments and highlight the critical importance of vegetation patches serving as refuge cover.

## Graphical abstract

This study, for the first time, reveals the individual variability in wolf responses to human disturbance. By fitting Hidden Markov Models to data from GPS‐GSM collars deployed on 26 wolves, we found significant variation between individuals in their responses to different levels of anthropogenic pressure and across different times of day, highlighting a non‐uniform strategy for coping with perturbations in human‐dominated landscapes. Our findings underscore the diverse behavioural adjustments employed by wolves to persist in these environments and highlight the critical importance of vegetation patches serving as refuge cover.

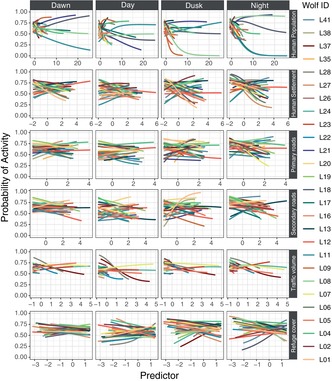

## 1 Introduction

A large variability of activity patterns has been reported in mammals and, particularly, in carnivores (Bennie et al. 2014). This variability underscores the adaptable nature of behavioral reactions to diverse ecological pressures, ultimately setting context‐dependency in the diel cycle use among species and even populations (Ferreiro‐Arias et al. 2021). Consequently, this adaptability in diverse mammalian species can yield substantial disparities in activity profiles among populations across different ecological contexts (Bennie et al. 2014; Ensing et al. 2014; Ferreiro‐Arias et al. 2021; Gaynor et al. 2018).

In the case of wolves (*Canis lupus*), several studies reported contrasted activity patterns across their distribution range, displaying diurnal, crepuscular nocturnal, or bimodal activity (Ciucci et al. 1997; Eriksen et al. 2011; Mech 1992; Reichmann and Saltz 2005). Among these different ecological settings, several factors have been pinpointed to explain the prevailing activity patterns. Consequently, periods when wolves engage in activity may fluctuate due to factors including sex, age, physiological, and social status (Eggermann et al. 2009; Jedrzejewski et al. 2001; Theuerkauf et al. 2003) as well as across distinct reproductive seasons (e.g., mating or pup‐rearing) (Rio‐Maior et al. 2018; Theuerkauf et al. 2003; Tsunoda et al. 2009). Beyond these intrinsic factors, an array of extrinsic influences also contributes to the variability in activity rhythms. External factors, such as prey availability (Ballard et al. 1991; Mech and Merrill 1998), human activity (Ciucci et al. 1997; Mancinelli et al. 2019), or even weather and moon phases may contribute to explain fluctuations in wolf activity (Fancy and Ballard 1995; Kolenosky and Johnston 1967; Mech and Cluff 2011).

Although the factors mentioned previously have an impact on wolf activity, the shift from diurnal to nocturnal habits appears to be primarily driven by localized levels of human activity in temperate regions (Theuerkauf 2009; Martínez‐Abraín et al. 2023). Several studies showed that wolves are able to perceive the negative risk related to human‐induced mortality, being able to avoid times and places where the risk associated with human encounters is highest (Carricondo‐Sanchez et al. 2020; Llaneza et al. 2016; McNay 2002; Mech and Cluff 2011; Sazatornil et al. 2016; and Theuerkauf, Jȩdrzejewski, Schmidt and Gula 2003). However, several studies have reported that wolves may exhibit contrasted and ambivalent behavioral responses towards anthropogenic disturbance (Dennehy, Llaneza, and López‐Bao 2021; Martínez‐Abraín et al. 2023; Zimmermann et al. 2014). This variability suggests a tendency of wolves to adjust their behavior based on the degree of human presence rather than differences in infrastructure densities (i.e., settlements or roads), which may hold significance when persevering in landscapes heavily influenced by humans (Dennehy, Llaneza, and López‐Bao 2021; Llaneza et al. 2016; Sazatornil et al. 2016). Thus, such behavioral flexibility would imply that behavioral adjustments exhibited by wolves in response to distinct ecological conditions could potentially lead to highly variable behaviors among populations and even individuals.

Nonetheless, the studies reporting these behavioral adaptations and avoidance patterns often fall short of thoroughly exploring the individual diversity that underlies such responses (Ciucci et al. 1997; Kusak, Skrbinšek, and Huber 2005; Mancinelli et al. 2019; Theuerkauf, Jȩdrzejewski, Schmidt and Gula 2003; Theuerkauf et al. 2003; Zimmermann et al. 2014). Different individuals can manifest diverse responses to human presence influenced by their habituation to humans (Carricondo‐Sanchez et al. 2020; McNay 2002), but few studies have fully explored wolf inter‐individual variation in avoidance of human disturbance, limiting the inference to a consistent spatial avoidance of human infrastructure mostly at the population level (Carricondo‐Sanchez et al. 2020). Other studies pointed out that disparities in the way wolves avoid human presence might stem from actual distinctions in personality, potentially shaped during their early years (Milleret et al. 2019; Sanz‐Pérez et al. 2018). This suggestion stems from a connection between the attributes of wolves' natal territories and their habitat preferences in adulthood. Notably, Scandinavian wolf pairs displayed a tendency to utilize areas in proximity to humans less frequently when their natal territories exhibited higher levels of human encroachment, which stands in contrast with wolves born in regions with a lower degree of anthropogenic influence (Milleret et al. 2019). Hence, in human‐dominated landscapes areas where wolves have managed to persist, they may have adapted their behavior to minimize encounters with humans by exhibiting avoidance patterns and increased wariness toward areas with high human activity.

In this study, we took advantage of a wolf population persisting in a highly human‐dominated landscape in the NW of the Iberian Peninsula, after a long period of intense human persecution to explore how wolf activity is influenced by humans and to investigate the individual variability in temporal avoidance responses of wolves toward different types of anthropogenic disturbance. Iberian wolves are suitable for exploring the impact of human disturbance on individual variation of wolf activity. The life history of the Iberian wolves is characterized by a historical and intense human persecution, which is epitomized in *ca*. 15,000 wolves estimated to be killed in just a 5‐year period during the 19th century (Rico and Torrente 2000). Systematic persecution preceded a considerable contraction of wolf range in Iberia (Clavero et al. 2022; Nores and López‐Bao 2022; Valverde 1971). After an intense period of persecution around the mid‐20th century, only two wolf populations remained during the 1970s: the large north‐western Iberian wolf population (shared with Portugal) (Chapron et al. 2014; Valverde 1971), and three small nuclei in the Sierra Morena area, all of them now extinct (López‐Bao et al. 2018). After this bottleneck, evidence suggest a decrease in the genetic diversity of the Iberian wolf population (Lobo, López‐Bao, and Godinho 2023; Salado et al. 2022). Hence, it is expected that this historical persecution could have profoundly influenced the behavior and ecology of the wolves persisting in these human‐dominated landscapes (Sazatornil et al. 2016).

## 2 Materials and Methods

### 2.1 Study Area

**FIGURE 1:**
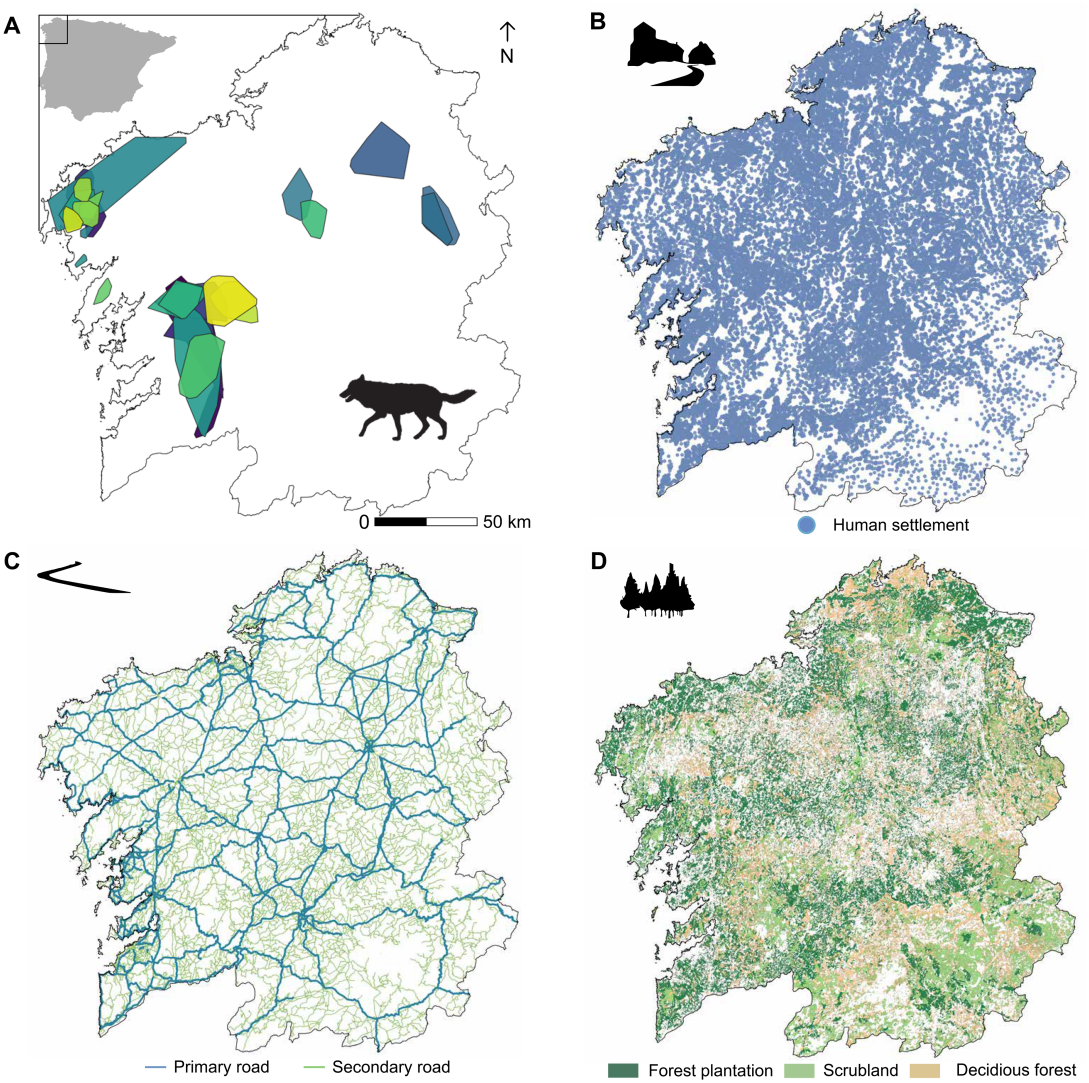
Study area showing the location of Galicia within Iberian Peninsula and the distribution of home ranges of the wolves monitored in this study (A), the spatial distribution of human settlements (B), paved roads (C), and refuge cover (D).

This study was carried out in Galicia (NW Spain; *ca*. 30,000 km^2^), where wolves have persisted continuously over the past two centuries (Clavero et al. 2022; Núñez‐Quirós, García‐Lavandera, and Llaneza 2007). Over recent decades, Galician landscapes have undergone a transformation from predominantly agricultural terrain to landscapes characterized by extensive pine and eucalyptus forest plantations (Calvo‐Iglesias, Fra‐Paleo, and Diaz‐Varela 2009). This transformation was accompanied by a proliferation of human infrastructures in both rural and natural settings, contributing to landscapes that are profoundly shaped by human activity (Llaneza, López‐Bao, and Sazatornil 2012). Besides forest plantations, the landscape is also characterized by pastures for livestock rearing, both covering ca. 55% of the region. Native scrublands and deciduous forests cover ca. 17% and 10%, respectively (SIOSE National Technical Team 2022). Galicia is characterized by a high human population density (93.7 inhabitants/km^2^), but human settlements are widely dispersed (*ca*. 3 human settlements/km^2^) with an average paved road density of 3.5 km/km^2^ (INE 2010) (Figure 1). Wolves occupy most of Galicia (91% of Galicia estimated in 2021–2022) and show a stable trend in the last decade (2013–2015 vs. 2021–2022), based on estimates of breeding packs (Llaneza et al. 2015, 2022).

### 2.2 Wolf Captures and Data Collection

Data from this study comes from a sample of 26 wolves captured and monitored between 2006 and 2014. Sex and age were determined in situ (*n*_males_ = 14, *n*_females_ = 12). Age was determined by assessing the dental pattern and individuals were classified as subadults (1–2 years, *n* = 19) or adults (≥ 2 years, *n* = 10) (Gipson et al. 2000). Wolves were also classified as pack members (*n* = 22) and non‐pack members (dispersers and lone wolves, *n* = 6). To classify wolves as pack and non‐pack members, we investigated the movements of wolves in relation to the location of den and rendezvous sites. We adhered to established protocols employing howling points utilized for wolf monitoring in the Iberian Peninsula (Llaneza, García, and López‐Bao 2014; Palacios et al. 2016). Additionally, we integrated data on the location of these sites with occasional visual observations of both collared and non‐collared wolves. Wolves that exhibited repeated presence near a specific den or rendezvous site with pups, or were observed with other pack members or pups, were classified as pack members. Wolves L04, L21 and L43 were first classified as pack members and left their pack during monitoring (Table S1). Wolves were monitored with GPS–GSM collars (Tellus T3H and T5H models from Followit, Sweden). GPS–GSM collars were scheduled in order to record wolf locations with a frequency of 2 h. Wolves included in this study were captured with Belisle leg‐hold snares (Edouard Belisle, Saint Veronique, PQ, Canada) and chemically immobilized by intramuscular injection of 0.10 mg/kg of medetomidine (Dormitor, Merial, Lyon, France). Capture procedures during fieldwork were adhered to animal welfare regulation under the permits 19/2006, 71/2009, 86/2011, and 95/2013 from the Regional Government of Galicia (Xunta de Galicia). Fieldwork procedures were adhered to regulations of animal welfare (Spanish Decree 53/2013). Immobilization was reversed by the intramuscular injection of atipamezole (Revertor, Merial, Lyon, France). Captured individuals were evaluated as clinically healthy at the moment of capture, showing only minor lesions associated with trapping (e.g., skin abrasion).

For each location, we extracted information of date and time from GPS–GSM collars and sunrise and sunset times from the Spanish National Geographical Institute (https://astronomia.ign.es/). In order to better classify activity records into nocturnal or diurnal behaviors, we subdivided a 24‐h period into 4 classes: dawn (1 h before and after sunrise); day (1 h after sunrise until 1 h before sunset); dusk (1 h before and after sunset); and night (1 h after sunset and 1 h before sunrise).

### 2.3 Inferring Wolf's Activity through Hidden Markov Models

We used hidden Markov models (hereafter, HMMs) to infer the underlying behavioral states (inactive or active) for each location. We first split the tracks of each wolf every time locations were missed and periods between consecutive recordings exceeded 4 h (Michelot, Langrock, and Patterson 2016). Then we fitted two‐state HMMs considering successive time series of locations to calculate the step length and the turning angle at each time point (Michelot, Langrock, and Patterson 2016). Step lengths were defined as the distance between consecutive locations while turning angles were defined as the magnitude of changes in direction between two successive steps. Step lengths were fitted with a Gamma distribution with a mean step length to 0.1 for inactive behaviors and 1 for active behaviors, with a corresponding standard deviation of 0.1 and 1, respectively. We further included a zero‐mass parameter to deal with a zero‐inflated distribution of step lengths (Michelot, Langrock, and Patterson 2016). We fitted turning angles with a von Mises distribution, with a mean turning angle and angle concentration of 3.14 (i.e., *pi*) and 0.5 for inactive states and 0 and 5 for active states, respectively. Besides step lengths and turning angles, we have included accelerometer data as a third data stream on HMMs to better define “active” and “inactive” behavioral states (Conners et al. 2021; McClintock and Michelot 2018). We included the sum of the X‐axis and Y‐axis values recorded by the accelerometer at the time each GPS coordinate was logged, as it provides reliable measures of activity which complements step lengths and turning angles (Petroelje et al. 2020). Since accelerometer loggers provide only positive and discrete values, we employed a negative binomial distribution with a size parameter of 0.01 for inactive states and 30 for active states, and a probability parameter of 0.8 for inactive states and 0.3 for active states. We expected to find an “active” state characterized by long step lengths, turning angles close to zero indicating directionality in the movement and large accelerometer values. Conversely, we anticipated an “inactive” state to reflect considerably short step lengths among successive locations, large turning angles, and accelerometer values close to zero (Franke et al. 2006; Ylitalo, Heikkinen, and Kojola 2021).

In order to fit a HMM, the likelihood function, which gauges the plausibility of the observed data given a set of parameter values, must be numerically optimized (Michelot, Langrock, and Patterson 2016). To minimize the negative log‐likelihood in our HMMs, we utilized the argument “*retryFits*” of the *fitHMM()* function set to 30 attempts, which introduces random perturbations of the parameter estimates at the current minimum (McClintock and Michelot 2018). The best model among these 30 attempts was selected by identifying the one with the highest maximum likelihood. HMMs were fitted by employing the “*momentuHMM*” package (McClintock and Michelot 2018) in R Statistical Software (R Core Team 2022).

### 2.4 Spatial Data and Covariates

We extracted values of different proxies of human disturbance. At fine scale (1 km^2^), we extracted information of human population density and distances from wolf locations to the nearest human settlement, primary and secondary roads, as well as refuge cover. We accomplished this by creating raster layers with varying resolution for each predictor, utilizing data resources available through the Spatial Data Infrastructure of Galicia (IDEG, http://mapas.xunta.gal/ideg). We further extracted information at the home‐range scale (i.e., home range) regarding the availability of refuge cover and the hourly average traffic volume. To this end, we extracted the percentage of refuge cover and traffic volume within a convex hull estimated based on a kernel density approach using the 90% of locations of each individual (Worton 1989).

We estimated the availability of land use cover supposedly acting as a functional refuge for wolves at both fine and large scale to test its influence on the activity patterns of the species. We defined refuge cover as the proportion of the area occupied by vegetation cover that could provide shelter for wolves (i.e., dense scrublands mainly represented by *Erica spp*. and *Ulex spp*., tree plantations and deciduous forests). We have extracted the size of patches of plantations, forests, dense scrublands and other land use classes that could provide shelter for wolves from land use layers available from the Spanish Land Occupancy Information System (SIOSE National Technical Team 2022). Then, we have rasterized the spatial layers with a resolution of 10 × 10 m and calculated the proportion occupied by refuge patches available within a 1 km radius for each of the obtained locations as well as for the convex hull estimated from the 90% of locations. To this end, we used the R package “*landscapemetrics*” (Hesselbarth et al. 2019).

In order to estimate the level of human activity at large scale, we employed as a proxy the hourly average intensity (HAI) of traffic available in the Annual Traffic Reports from the Department of Infrastructure and Mobility of Xunta de Galicia (https://infraestruturasemobilidade.xunta.gal/portada). These detailed reports provide the average hourly traffic volume per road section for each day of the week of all months, from 1993 to 2021. We extracted the road segments occurring in each wolf’ home ranges and subsequently the traffic volume corresponding to the time and date of each wolf locations. Then, we computed the total hourly traffic volume by summing the total records of each road section occurring within the estimated convex hull.

To extract values of human population density, we used data containing the validated location of > 30,000 human settlements along with their population size in 2010, resulting in a fine‐scale and robust characterization of the large dispersion of inhabitants in the region. We used the “*Kernel Density*” tool in ArcGIS Pro software (Environmental Systems Research Institute 2023), leveraging the previously mentioned shapefile layer containing the locations of the population entities. In this process, we assigned weight to each point based on the population size of the corresponding entity. This resulted in a 1 × 1 km raster of the estimated human population size per square kilometer. Subsequently, we assigned the value of human population density to each wolf locations included in that cell.

To extract values of Euclidean distances to human settlements, primary and secondary roads, we built up three 10 × 10 m raster layers depicting the Euclidean distance of the centre of each raster cell to the nearest human infrastructure by using the “*Euclidean Distance”* tool from ArcGIS Pro software (Environmental Systems Research Institute 2023). For paved roads, we first reclassified roads as primary and secondary based on traffic intensity, fencing and number of lanes (Dennehy, Llaneza, and López‐Bao 2021) and, subsequently, calculated the distance of the centre of each cell to the nearest line feature. For each human infrastructure, we assigned the value of Euclidean distance to each wolf locations included in that cell.

### 2.5 Estimating the Effects of Anthropogenic Disturbance on Wolf Activity

To determine the main factors related to anthropogenic disturbance driving changes between “active” and “inactive” behavioral states, we used a set of predictors as covariates in HMMs (see *Spatial data and covariates* section). Instead of merging all predictors in a set of different plausible models and to perform model selection based on information criteria (e.g., AIC or BIC), we relied on a set of a priori hypotheses to construct the fixed structure of our models and test the influence of different drivers of anthropogenic disturbance (see Table S2 for a detailed description of predictors and hypotheses). Thus, we separately included interactions between the time of day (i.e., dawn, day, dusk, and night) and the following predictors as covariates: fine‐scale refuge cover, human population density, hourly traffic volume, Euclidean distance to the nearest human settlement, and distances to the nearest primary and secondary roads. Lastly, we further included an interaction term between human population density and refuge cover at home‐range scale to test if vegetation cover may buffer the effects of human population density on overall wolf activity. Continuous predictors were previously scaled and centred in order to avoid convergence issues when running models. We further tested the multicollinearity of the predictors by calculating variance inflation factors (VIF) and pairwise Pearson correlation coefficients using the *car* and *corrplot* packages, respectively (Fox et al. 2007; Wei et al. 2017) (Figure S1). Wolf L43 was not included for modeling analysis since we could not estimate several predictor values.

### 2.6 Analyzing the Inter‐Individual Variability of Wolf Responses

To investigate the effects of anthropogenic disturbance predictors on wolf behavior, we fitted several HMMs. Initially, we fit a population‐level HMM using data from all wolves to estimate a single set of parameters that represent the average relationships between predictors and wolf behavior across the entire population. For a detailed analysis of individual variability, we applied an individual‐based approach by fitting separate HMMs for each wolf. Each individual HMM retained the same covariates as specified by our a priori hypotheses (Table S2). This method allows for the estimation of individual‐specific coefficients for each predictor, thereby capturing unique responses of each wolf to each proxy of anthropogenic disturbance. Since this approach does not guarantee consistency in behavioral state classification across individuals, we have visually inspected the derived state‐dependent distributions for each data stream and each individual. However, as our aim is solely to classify two behavioral states (i.e., active vs. inactive), we argue that any variability observed does not present a significant obstacle to the accurate inference and interpretation of our results. Hence, the stationary probabilities from the population‐level HMM would reflect general trends and responses across the population. In contrast, stationary probabilities from each individual HMM would reveal how specific wolves respond to various predictors, enabling an examination of both population‐wide patterns and individual‐specific behaviors. To extract and visualize the stationary probabilities, we used the *plotStationary()* function from the “*momentuHMM*” package (McClintock and Michelot 2018). In order to estimate conditional stationary probabilities of activity, we fixed the values of all continuous predictors at their median values. For categorical predictors, we maintained the reference level of the factor to isolate the effect of the predictor of interest. For specific visualization of individual distributions of activity across different day periods, continuous predictors were further categorized into three classes: low, medium, and high, which were defined based on the first quartile, median, and third quartile values, respectively. Model visualization was carried out using the “*ggplot2*” package (Wickham 2016). All analyses were carried out in R Statistical Software v. 4.2.2 (R Core Team 2022).

## 3 Results

GPS–GSM devices recorded tracks spanning a total of 54,721 wolf locations from 26 individuals, covering approximately 109,442 h of sampling. Mean (± SD) of sampling days per individual was 193 (± 100) days (range: 52–397) (Table S1). Mean (± SD) percentage of missing locations per individual was 12.5% (± 16.9%) (Table S1).

### 3.1 State‐Dependent Parameters and Probabilities

Splitting our dataset into complete tracks resulted in a ~10% reduction in locations available for HMMs. HMMs converged successfully in all models across the 30 attempts, reaching consensus on state‐dependent parameters. For population‐level HMM, the mean (± SD) of step length and the mean (and concentration) of turning angles for inactive locations were 0.03 (± 0.03) km and − 3.14 rad (0.5), respectively (Figure S2). For active behaviors, the mean (± SD) of step length and the mean (and concentration) of turning angles were 1.45 (± 1.63) km and 0.12 rad (0.12), respectively (Figure S2). The zero‐mass parameters for step length were 3.5 × 10^−5^ for inactive and 3.1 × 10^−4^ for active states. Additionally, the mean (± SD) of the sum of dual axis accelerometer values for inactive states was 3.32 (± 0.09) and for active states was 25.31 (± 0.36), respectively (Figure S2).

State transition probabilities based on mean covariates values indicates that the probability of wolves remaining in the inactive state is 0.87, while transitioning to the active state is 0.13. Conversely, the probability of remaining in the active state is 0.36, while transitioning to the inactive state is 0.64. Stationary‐state distributions of step lengths and acceleration largely differ between behavioral states, indicating a clear separation of behavioral states in function of variations in signal patterns obtained from accelerometer devices and distances between consecutive locations (Figure S2). Turning angles showed flat density distributions in both states, implying random movement directions (Figure S2).

### 3.2 Temporal Responses Toward Anthropogenic Disturbance

**FIGURE 2:**
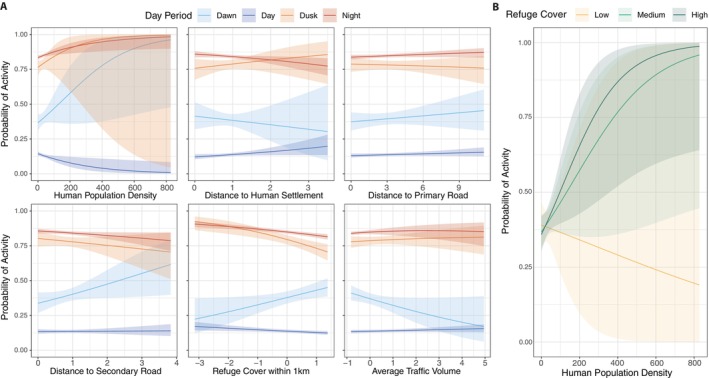
Population‐level effects of anthropogenic disturbance predictors on wolf activity. Panel A shows the stationary probability of activity as a function of the interaction between various predictors and the period of the day. Panel B illustrates the interaction between human population density and refuge cover at the home range scale on overall wolf activity. Refuge cover was classified as low, medium, and high based on the first quartile, median, and third quartile values, respectively. Shaded regions along each line denote the corresponding 95% confidence intervals.

Overall, for most predictors related to anthropogenic disturbance, we observed either a lack of effects or weak trends at population level on the probability of activity during night, dusk, and daylight periods (Figure 2A). We found positive trends at dawn for human population density, distances to primary and secondary roads, and fine‐scale refuge cover (Figure 2A). Conversely, we found negative trends at dawn for distances to human settlements and traffic volume (Figure 2A). Additionally, there was a weak trend for wolves to reduce their diurnal activity as human population density increased and the distance to human settlements decreased (Figure 2A). In contrast, we observed a clear effect in the interaction between human population density and the amount of refuge cover at the home range scale on overall wolf activity (Figure 2B). When refuge cover is high or medium at the home range scale, the probability of activity increases despite higher human population density. Conversely, when refuge cover is low, the probability of activity decreases drastically as human population density increases (Figure 2B).

### 3.3 Inter‐Individual Variability within Temporal Avoidance Patterns

**FIGURE 3:**
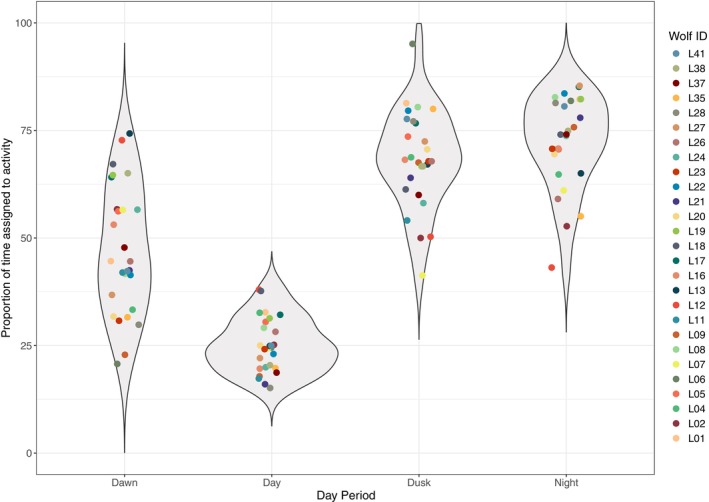
Proportion of time assigned to activity across different day periods. Violin plots for each day period illustrate the variability within the wolf population in the proportion of time that individuals are active. Colored dots illustrate the individual‐specific values of proportion of time expended being active.

Wolves exhibited clear inter‐individual differences in the proportions of time they were active during different periods of the day (Figure 3). At the population level, wolves showed a higher proportion of time being active during the night and dusk compared to dawn or day periods. We found considerable inter‐individual variability at in the proportion of time active at dawn, ranging from approximately 23% to 75% of the time, which drastically decreased during daylight hours (Figure 3). Conversely, moderate inter‐individual variability in the proportion of time spent active was observed during twilight and night (Figure 3).

**FIGURE 4:**
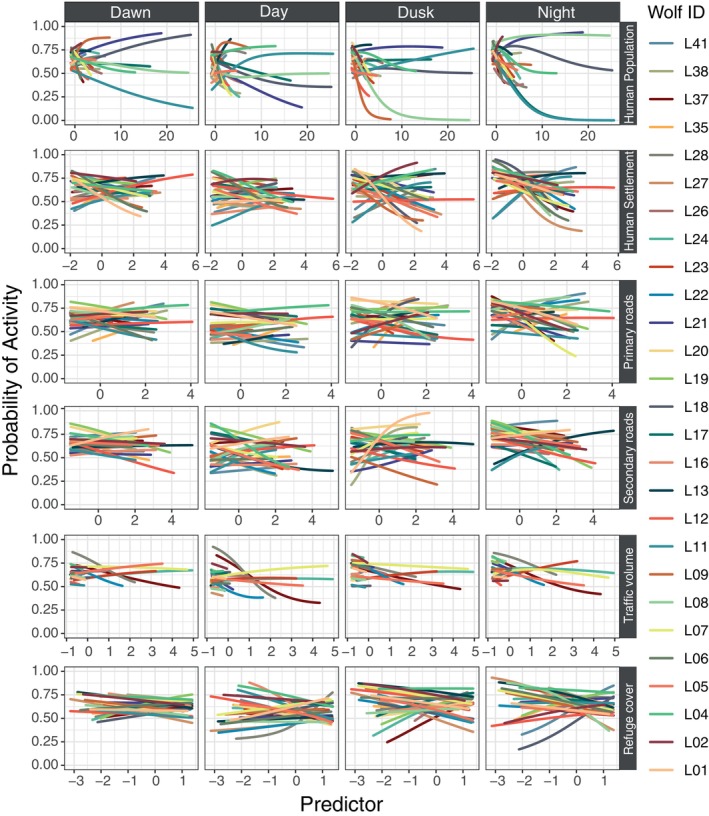
Individual responses of wolves to anthropogenic disturbance. Each column displays a different period of the day. Each row, listed from top to bottom, represents a different predictor related to anthropogenic disturbance: Human population density, distance to human settlements, distance to primary roads, distance to secondary roads, hourly traffic volume, and refuge cover at fine scale. Colored lines within each panel represent the responses of individual wolves to the respective predictor.

**FIGURE 5:**
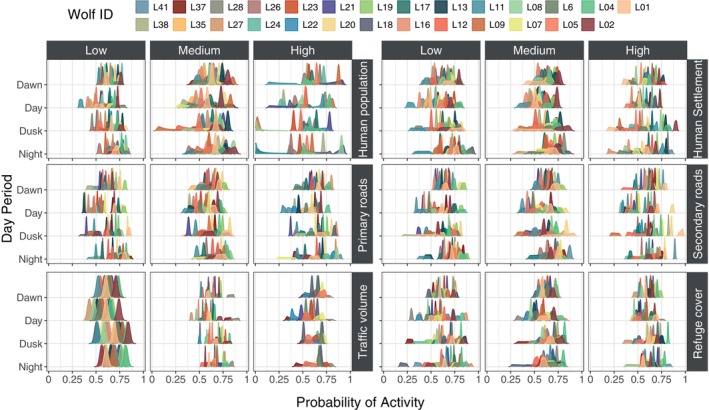
Ridge density plot showing individual distributions of probability of activity and the inter‐individual differences across levels of anthropogenic disturbance and day periods. The y‐axis represents the period of the day, and the x‐axis shows the probability of activity. The plot is organized into six columns (three categories of respective predictor values: low, medium, and high) and three rows: The first row displays human population density (left) and distance to human settlements (right), the second row shows distance to primary roads (left) and distance to secondary roads (right), and the third row presents hourly traffic volume (left) and refuge cover at fine scale (right). Each density function is colored by individual wolf ID. Thicker density functions indicate higher individual variability, while narrower, more peaked functions suggest lower variability. Greater overlap between density functions indicates less inter‐individual variability, whereas less overlap indicates higher variability for a given predictor level during a specific period of the day.

Overall, we found that individual responses to various predictors of anthropogenic disturbance were highly diverse, showing both negative and positive effects depending on the predictor and the period of the day (Figure 4). Specifically, individual responses to human population density exhibited substantial variability. At high population densities, both individual and inter‐individual variability in the probability of wolf activity increased markedly, with some wolves demonstrating pronounced negative trends while others showed positive trends, even during daylight hours (Figures 4 and 5). In contrast, when human population density was medium or low, variability in responses decreased significantly (Figure 5). For distances to human settlements, primary roads, and secondary roads, we observed similar ambivalent responses among wolves (Figure 4). Some wolves approached these features during various times of the day, whereas others ether displayed no effect (e.g., L12) or consistently avoided them (e.g., L27) (Figure 4). This pattern of variability was consistent across all predictor categories (low, medium, and high) and day periods (Figure 5). Regarding traffic volume, we found that both individual and inter‐individual variability were lower across all times of the day (Figures 4 and 5). Additionally, as the amount of refuge cover diminished, wolves displayed ambivalent responses during day, dusk, and night, with both positive and negative effects depending on the amount of available refuge cover (Figure 4). However, inter‐individual variability in activity decreased during dawn periods and increasing fine‐scale refuge cover availability (Figure 5).

## 4 Discussion

Our findings contribute to the understanding of how wolves adjust their behaviors to cope with human activity and highlight the inter‐individual variability in such responses. Consistent with previous studies, our results reinforce the idea that, in human‐dominated landscapes wolves are predominantly nocturnal in order to reduce exposure risk to humans. (Ciucci et al. 1997; Kusak, Skrbinšek, and Huber 2005; Mancinelli et al. 2019; Vilà 1995). This is in line with the concept of temporal avoidance, where wolves adjust their behavior to avoid periods of high human activity, reducing the risk of human‐induced mortality (Gaynor et al. 2018; Kronfeld‐Schor and Dayan 2003). As expected, higher human population density was associated with reduced daytime activity, emphasizing the wolves' sensitivity to human presence. The negative effects on daytime activity were more pronounced with increasing proximity to human settlements. Conversely, we found a positive effect of human population density on nocturnal activity, dusk and dawn, thus indicating that wolves may utilize these times to venture closer to such areas when the human presence is lower. This behavior is consistent with a spatio‐temporal avoidance patterns engaged by the notion that wolves actively avoid areas with higher human activity to reduce the risk of encounter with humans (Mancinelli et al. 2019; Theuerkauf, Jȩdrzejewski, Schmidt, and Gula 2003). Similarly, recent studies also showed that Iberian wolves avoided certain periods of the day for crossing different classes of roads because traffic flow was at its highest, reinforcing the idea that paved roads influence wolf behavior due to traffic volume (i.e., human activity) rather than the occurrence of roads per se (Dennehy, Llaneza, and López‐Bao 2021).

Refuge cover, represented by vegetation providing shelter for wolves, also played a role in modulating activity by hampering the effects of human perturbation. Higher levels of refuge cover at fine scale were slightly associated with reduced nocturnal and crepuscular activity and increased activity during dawn, indicating that areas with more shelter might encourage wolves to be more active during this period. In contrast, we observed a clear interaction effect between human population density and the amount of refuge cover at the home range scale on overall wolf activity. This finding underscores the importance of large‐scale habitat features in buffering the adverse effects of human presence. While fine‐scale refuge cover showed variable and individual‐specific effects, it did not demonstrate such strong, consistent impacts at population level. This suggests that at a larger spatial scale, the availability of extensive refuge cover can play a critical role in allowing wolves to maintain their activity levels despite anthropogenic pressures. The ability of extensive refuge areas to mitigate the negative impacts of human density highlights the need for conservation strategies that focus on preserving and enhancing large, contiguous areas of vegetation functionally acting as refuge for wolves. This could be particularly vital in regions facing increasing human encroachment, where maintaining or restoring substantial refuge cover within wolf home ranges may be key to supporting their ecological resilience and behavioral adaptability (Grilo et al. 2019; Llaneza et al. 2016; Sazatornil et al. 2016). Hence, our results underscore the importance of maintaining patches of vegetation that can act as effective refuges for wolves from a functional point of view (Grilo et al. 2019; Llaneza et al. 2016; Sazatornil et al. 2016), in order to facilitate the persistence of wolves in human‐dominated landscapes.

One of the significant contributions of our study is the exploration, for the first time, of this inter‐individual variability in wolf responses toward anthropogenic disturbance. Remarkably, we observed a substantial individual variability in the temporal avoidance of wolves toward proxies of human disturbance that obscures clear population‐level effects, suggesting a non‐uniform strategy of wolves in adapting to human‐dominated environments. Overall, this variability masked the effects of predictors at population‐level, but there was particularly evident in responses to human population density, where some wolves exhibited clear negative trends in activity, while others showed positive trends, even during daylight hours. This highlights the complexity of wolf behavior and their capacity for individual‐specific adaptation. Furthermore, the ambivalent responses to distances from human settlements and roads indicate that wolves may employ diverse strategies, with some individuals approaching these features while others avoid them consistently. This pattern suggests that wolves are capable of behavioral plasticity, potentially adjusting their activity to balance the risks and benefits of human proximity. Hence, our comprehensive examination of predictors highlights the complex and variable nature of wolf responses to anthropogenic disturbances.

The strategies of temporal avoidance toward humans (i.e., increased nocturnality) has been pointed out as one of the main mechanisms driving human–carnivore coexistence, enhancing the persistence of large predators in human‐dominated landscapes (Lamb et al. 2020). Given the historical persecution of wolves in Iberian landscapes (Clavero et al. 2022; Nores and López‐Bao 2022; Núñez‐Quirós, García‐Lavandera, and Llaneza 2007; Rico and Torrente 2000), nocturnal behaviors may be favored from an adaptative perspective by reducing human‐induced mortality. Differences in individual responses and personalities in animal behavior can be explained by adaptive pressures (Dall, Houston, and McNamara 2004; Wilson 1998), which often results in polymorphic populations of risk‐taking and risk‐averse individuals (Bombieri et al. 2021; Wolf et al. 2007). This spectrum of behaviors can have systemic consequences on the overall fitness of individuals within a population (Smith and Blumstein 2008) and the existence of a gradient along the shyness‐boldness spectrum can become particularly pertinent in populations subjected to persecution. This can lead to subsequent changes in the equilibrium of behavior frequencies within populations, adding a nuanced layer to our understanding of the adaptive strategies employed by animals in response to external threats (Wolf et al. 2007). Hence, if along the life history of Iberian wolves an increased nocturnality and risk‐averse behaviors continued over multiple generations being favored by a reduced human‐induced mortality, it could potentially lead to changes in the behavior of wolves at population level (Martínez‐Abraín et al. 2023).

However, our understanding of the factors influencing behavioral traits is still quite limited. It is plausible that this tendency is an adaptive trait inherited through generations, possibly developed as a response to the selective pressure against behaviors that draw wolves toward human activities, a phenomenon already observed in other species (Agnvall et al. 2012; Carrete et al. 2016). Additionally, natal conditions could play a role in shaping this avoidance behavior and the individual variability in these spatio‐temporal responses (Carricondo‐Sanchez et al. 2020; Milleret et al. 2019). While learning before independence may contribute, there are indications that the fear of humans has a genetic influence (Carricondo‐Sanchez et al. 2020; Fox 1972; Hall et al. 2015; Milleret et al. 2019; Saetre et al. 2006). Given that a significant proportion of wolf mortality is linked to humans (Blanco and Cortés 2007; Rico and Torrente 2000), it is reasonable to argue that the ability to avoid humans could be an advantageous trait subject to human‐induced selection pressures. This behavior may manifest as an innate component (Saetre et al. 2006), learned from parents (Milleret et al. 2019), or a combination of both. However, establishing a direct relationship between avoidance patterns and specific environmental conditions is challenging, making it speculative to determine the true adaptiveness of observed behaviors. While we focused on analyzing temporal avoidance patterns and their inter‐individual variability, it is crucial to acknowledge these limitations, emphasizing the need for further research to validate these speculations. Future investigations exploring the connection between behavioral variability and genetic relatedness hold the potential to yield intriguing insights in the adaptability of wolves toward different ecological scenarios and human‐dominated landscapes.

## Author Contributions

**Iago Ferreiro‐Arias:** conceptualization (equal), data curation (lead), formal analysis (lead), investigation (lead), methodology (lead), resources (equal), software (lead), validation (lead), visualization (lead), writing – original draft (lead), writing – review and editing (lead). **Emilio José García:** methodology (supporting), resources (supporting), writing – review and editing (supporting). **Vicente Palacios:** methodology (supporting), resources (supporting), writing – review and editing (supporting). **Víctor Sazatornil:** methodology (supporting), resources (supporting), writing – review and editing (equal). **Alejandro Rodríguez:** methodology (supporting), resources (supporting), writing – review and editing (equal). **José Vicente López‐Bao:** methodology (supporting), resources (supporting), writing – review and editing (supporting). **Luis Llaneza:** conceptualization (equal), funding acquisition (lead), methodology (lead), project administration (lead), resources (lead), supervision (lead), writing – review and editing (equal).

## Conflicts of Interest

The authors declare no conflicts of interest.

## Supporting information


Data S1.

## Data Availability

Data supporting the findings of this study are available on request from A.RE.NA. Asesores en Recursos Naturales, S.L. Data is not publicly available due to containing information of location of wolves and packs, a protected species under current Spanish legislation (Spanish Royal Decree 139/2011 and Ministerial Order TED/980/2021).

## References

[ece370397-bib-0001] Agnvall, B. , M. Jöngren , E. Strandberg , and P. Jensen . 2012. “Heritability and Genetic Correlations of Fear‐Related Behaviour in Red Junglefowl–Possible Implications for Early Domestication.” PLoS One 7, no. 4: e35162. 10.1371/journal.pone.0035162. 22536354 PMC3334967

[ece370397-bib-0002] Ballard, W. B. , L. Ayres , C. Gardner , and J. Foster . 1991. “Den Site Activity Patterns of Gray Wolves, *Canis lupus*, in Southcentral Alaska.” Canadian Field‐Naturalist 105, no. 4: 497–504.

[ece370397-bib-0003] Bennie, J. J. , J. P. Duffy , R. Inger , and K. J. Gaston . 2014. “Biogeography of Time Partitioning in Mammals.” Proceedings of the National Academy of Sciences 111, no. 38: 13727–13732. 10.1073/pnas.1216063110. PMC4183310 25225371

[ece370397-bib-0004] Blanco, J. C. , and Y. Cortés . 2007. “Dispersal Patterns, Social Structure and Mortality of Wolves Living in Agricultural Habitats in Spain.” Journal of Zoology 273, no. 1: 114–124. 10.1111/j.1469-7998.2007.00305.x.

[ece370397-bib-0005] Bombieri, G. , V. Penteriani , M. D. M. Delgado , C. Groff , L. Pedrotti , and K. Jerina . 2021. “Towards Understanding Bold Behaviour of Large Carnivores: The Case of Brown Bears in Human‐Modified Landscapes.” Animal Conservation 24, no. 5: 783–797. 10.1111/acv.12680.

[ece370397-bib-0006] Calvo‐Iglesias, M. S. , U. Fra‐Paleo , and R. A. Diaz‐Varela . 2009. “Changes in Farming System and Population as Drivers of Land Cover and Landscape Dynamics: The Case of Enclosed and Semi‐Openfield Systems in Northern Galicia (Spain).” Landscape and Urban Planning 90, no. 3: 168–177. 10.1016/j.landurbplan.2008.10.025.

[ece370397-bib-0007] Carrete, M. , J. Martínez‐Padilla , S. Rodríguez‐Martínez , N. Rebolo‐Ifrán , A. Palma , and J. L. Tella . 2016. “Heritability of Fear of Humans in Urban and Rural Populations of a Bird Species.” Scientific Reports 6, no. 1: 1–6. 10.1038/srep31060. PMC4976307 27499420

[ece370397-bib-0008] Carricondo‐Sanchez, D. , B. Zimmermann , P. Wabakken , et al. 2020. “Wolves at the Door? Factors Influencing the Individual Behavior of Wolves in Relation to Anthropogenic Features.” Biological Conservation 244: 108514. 10.1016/j.biocon.2020.108514.

[ece370397-bib-0009] Chapron, G. , P. Kaczensky , J. D. C. Linnell , et al. 2014. “Recovery of Large Carnivores in Europe's Modern Human‐Dominated Landscapes.” Science 346, no. 6216: 1517–1519. 10.1126/science.1257553. 25525247

[ece370397-bib-0010] Ciucci, P. , L. Boitani , F. Francisci , and G. Andreoli . 1997. “Home Range, Activity and Movements of a Wolf Pack in Central Italy.” Journal of Zoology 243, no. 4: 803–819. 10.1111/j.1469-7998.1997.tb01977.x.

[ece370397-bib-0011] Clavero, M. , A. García‐Reyes , A. Fernández‐Gil , E. Revilla , and N. Fernández . 2022. “On the Misuse of Historical Data to Set Conservation Baselines: Wolves in Spain as an Example.” Biological Conservation 276: 109810.

[ece370397-bib-0012] Conners, M. G. , T. Michelot , E. I. Heywood , et al. 2021. “Hidden Markov Models Identify Major Movement Modes in Accelerometer and Magnetometer Data From Four Albatross Species.” Movement Ecology 9, no. 1: 1–16. 10.1186/s40462-021-00243-z PMC7901071 33618773

[ece370397-bib-0013] Dall, S. R. X. , A. I. Houston , and J. M. McNamara . 2004. “The Behavioural Ecology of Personality: Consistent Individual Differences From an Adaptive Perspective.” Ecology Letters 7, no. 8: 734–739. 10.1111/j.1461-0248.2004.00618.x.

[ece370397-bib-0014] Dennehy, E. , L. Llaneza , and J. V. López‐Bao . 2021. “Contrasting Wolf Responses to Different Paved Roads and Traffic Volume Levels.” Biodiversity and Conservation 30, no. 11: 3133–3150.

[ece370397-bib-0016] Eggermann, J. , R. Gula , B. Pirga , et al. 2009. “Daily and Seasonal Variation in Wolf Activity in the Bieszczady Mountains, SE Poland.” Mammalian Biology 74, no. 2: 159–163. 10.1016/j.mambio.2008.05.010.

[ece370397-bib-0017] Ensing, E. P. , S. Ciuti , F. A. de Wijs , et al. 2014. “GPS Based Daily Activity Patterns in European Red Deer and North American Elk (*Cervus elaphus*): Indication for a Weak Circadian Clock in Ungulates.” PLoS One 9, no. 9: e106997. 10.1371/journal.pone.0106997. 25208246 PMC4160215

[ece370397-bib-0018] Environmental Systems Research Institute . 2023. ArcGIS Pro (Version 2.9) [Computer Software]. Redlands, CA: Environmental Systems Research Institute.

[ece370397-bib-0019] Eriksen, A. , P. Wabakken , B. Zimmermann , et al. 2011. “Activity Patterns of Predator and Prey: A Simultaneous Study of GPS‐Collared Wolves and Moose.” Animal Behaviour 81, no. 2: 423–431. 10.1016/j.anbehav.2010.11.011.

[ece370397-bib-0020] Fancy, S. G. , and W. B. Ballard . 1995. “Monitoring Wolf Activity by Satellite.” In Ecology and Conservation of Wolves in a Changing World, edited by L. N. Carbyn , S. H. Fritts , and D. R. Seip , 329–333. Edmonton, Alberta: Occasional Publication.

[ece370397-bib-0021] Ferreiro‐Arias, I. , J. Isla , P. Jordano , and A. Benítez‐López . 2021. “Fine‐Scale Coexistence Between Mediterranean Mesocarnivores Is Mediated by Spatial, Temporal, and Trophic Resource Partitioning.” Ecology and Evolution 11, no. 22: 15520–15533. 10.1002/ece3.8077. 34824772 PMC8601891

[ece370397-bib-0022] Fox, J. , G. G. Friendly , S. Graves , et al. 2007. “The Car Package.” R Foundation for Statistical Computing 1109: 1431.

[ece370397-bib-0023] Fox, M. W. 1972. “Socio‐Ecological Implications of Individual Differences in Wolf Litters: A Developmental and Evolutionary Perspective.” Behaviour 41, no. 3–4: 298–313. 10.1163/156853972X00077.

[ece370397-bib-0024] Franke, A. , T. Caelli , G. Kuzyk , and R. J. Hudson . 2006. “Prediction of Wolf (*Canis lupus*) Kill‐Sites Using Hidden Markov Models.” Ecological Modelling 197, no. 1: 237–246. 10.1016/j.ecolmodel.2006.02.043.

[ece370397-bib-0025] Gaynor, K. M. , C. E. Hojnowski , N. H. Carter , and J. S. Brashares . 2018. “The Influence of Human Disturbance on Wildlife Nocturnality.” Science 360, no. 6394: 1232–1235. 10.1126/science.aar7121. 29903973

[ece370397-bib-0026] Gipson, P. S. , W. B. Ballard , R. M. Nowak , and L. D. Mech . 2000. “Accuracy and Precision of Estimating Age of Gray Wolves by Tooth Wear.” Journal of Wildlife Management 64, no. 3: 752. 10.2307/3802745.

[ece370397-bib-0027] Grilo, C. , P. M. Lucas , A. Fernández‐Gil , et al. 2019. “Refuge as Major Habitat Driver for Wolf Presence in Human‐Modified Landscapes.” Animal Conservation 22, no. 1: 59–71. 10.1111/acv.12435.

[ece370397-bib-0028] Hall, N. J. , K. Lord , A.‐M. K. Arnold , C. D. L. Wynne , and M. A. R. Udell . 2015. “Assessment of Attachment Behaviour to Human Caregivers in Wolf Pups (*Canis lupus lupus*).” Behavioural Processes 110: 15–21. 10.1016/j.beproc.2014.11.005. 25447510

[ece370397-bib-0029] Hesselbarth, M. H. K. , M. Sciaini , K. A. With , K. Wiegand , and J. Nowosad . 2019. “Landscapemetrics: An Open‐Source R Tool to Calculate Landscape Metrics.” Ecography 42, no. 10: 1648–1657. 10.1111/ecog.04617.

[ece370397-bib-0030] INE . 2010. Censo de Población y Vivienda [Dataset]. Madrid, Spain: Instituto Nacional de Estadistica.

[ece370397-bib-0031] Jedrzejewski, W. , K. Schmidt , J. Theuerkauf , B. Jedrzejewska , and H. Okarma . 2001. “Daily Movements and Territory Use by Radio‐Collared Wolves (*Canis lupus*) in Bialowieza Primeval Forest in Poland.” Canadian Journal of Zoology 79, no. 11: 1993–2004. 10.1139/z01-147.

[ece370397-bib-0032] Kolenosky, G. B. , and D. H. Johnston . 1967. “Radio‐Tracking Timber Wolves in Ontario.” American Zoologist 7, no. 2: 289–303.

[ece370397-bib-0033] Kronfeld‐Schor, N. , and T. Dayan . 2003. “Partitioning of Time as an Ecological Resource.” Annual Review of Ecology, Evolution, and Systematics 34, no. 1: 153–181. 10.1146/annurev.ecolsys.34.011802.132435.

[ece370397-bib-0034] Kusak, J. , A. M. Skrbinšek , and D. Huber . 2005. “Home Ranges, Movements, and Activity of Wolves (*Canis lupus*) in the Dalmatian Part of Dinarids, Croatia.” European Journal of Wildlife Research 51, no. 4: 254–262. 10.1007/s10344-005-0111-2.

[ece370397-bib-0035] Lamb, C. T. , A. T. Ford , B. N. McLellan , et al. 2020. “The Ecology of Human–Carnivore Coexistence.” Proceedings of the National Academy of Sciences 117, no. 30: 17876–17883. 10.1073/pnas.1922097117. PMC7395549 32632004

[ece370397-bib-0036] Llaneza, L. , E. J. García , and J. V. López‐Bao . 2014. “Intensity of Territorial Marking Predicts Wolf Reproduction: Implications for Wolf Monitoring.” PLoS One 9, no. 3: e93015. 24663068 10.1371/journal.pone.0093015 PMC3963981

[ece370397-bib-0037] Llaneza, L. , E. J. García , V. Palacios , and J. V. López‐Bao . 2015. Censo de Lobo Ibérico En Galicia, 61. Xunta de Galicia (Spanish Administrative Institution): Consellería de Medio Ambiente.

[ece370397-bib-0038] Llaneza, L. , E. J. García , V. Palacios , V. Sazatornil , and J. V. López‐Bao . 2016. “Resting in Risky Environments: The Importance of Cover for Wolves to Cope With Exposure Risk in Human‐Dominated Landscapes.” Biodiversity and Conservation 25: 1515–1528.

[ece370397-bib-0039] Llaneza, L. , J. V. López‐Bao , and V. Sazatornil . 2012. “Insights Into Wolf Presence in Human‐Dominated Landscapes: The Relative Role of Food Availability, Humans and Landscape Attributes.” Diversity and Distributions 18, no. 5: 459–469.

[ece370397-bib-0040] Llaneza, L. , V. Palacios , A. Marcos , et al. 2022. *Estudio poblacional del lobo ibérico en Galicia, 2021–2022*. Xunta de Galicia (Spanish Administrative Institution): Consellería de Medio Ambiente, Territorio e Vivenda.

[ece370397-bib-0041] Lobo, D. , J. V. López‐Bao , and R. Godinho . 2023. “The Population Bottleneck of the Iberian Wolf Impacted Genetic Diversity but Not Admixture With Domestic Dogs: A Temporal Genomic Approach.” Molecular Ecology 32, no. 22: 5986–5999. 37855673 10.1111/mec.17171

[ece370397-bib-0042] López‐Bao, J. V. , F. Fleurke , G. Chapron , and A. Trouwborst . 2018. “Legal Obligations Regarding Populations on the Verge of Extinction in Europe: Conservation, Restoration, Recolonization, Reintroduction.” Biological Conservation 227: 319–325.

[ece370397-bib-0043] Mancinelli, S. , M. Falco , L. Boitani , and P. Ciucci . 2019. “Social, Behavioural and Temporal Components of Wolf (*Canis lupus*) Responses to Anthropogenic Landscape Features in the Central Apennines, Italy.” Journal of Zoology 309, no. 2: 114–124. 10.1111/jzo.12708.

[ece370397-bib-0044] Martínez‐Abraín, A. , Á. Llinares , L. Llaneza , et al. 2023. “Increased Grey Wolf Diurnality in Southern Europe Under Human‐Restricted Conditions.” Journal of Mammalogy 104, no. 4: 846–854. 10.1093/jmammal/gyad003. 37545665 PMC10399918

[ece370397-bib-0045] McClintock, B. T. , and T. Michelot . 2018. “momentuHMM: R Package for Generalized Hidden Markov Models of Animal Movement.” Methods in Ecology and Evolution 9, no. 6: 1518–1530. 10.1111/2041-210X.12995.

[ece370397-bib-0046] McNay, M. E. 2002. “Wolf‐Human Interactions in Alaska and Canada: A Review of the Case History.” Wildlife Society Bulletin (1973–2006) 30, no. 3: 831–843.

[ece370397-bib-0047] Mech, L. D. 1992. “Daytime Activity of Wolves During Winter in Northeastern Minnesota.” Journal of Mammalogy 73, no. 3: 570–571. 10.2307/1382025.

[ece370397-bib-0048] Mech, L. D. , and H. D. Cluff . 2011. “Movements of Wolves at the Northern Extreme of the Species' Range, Including During Four Months of Darkness.” PLoS One 6, no. 10: e25328. 21991308 10.1371/journal.pone.0025328 PMC3186767

[ece370397-bib-0049] Mech, L. D. , and S. B. Merrill . 1998. “Daily Departure and Return Patterns of Wolves, *Canis lupus*, From a Den at 80 (Degree) N Latitude.” Canadian Field‐Naturalist 112, no. 3: 515–517.

[ece370397-bib-0050] Michelot, T. , R. Langrock , and T. A. Patterson . 2016. “moveHMM: An R Package for the Statistical Modelling of Animal Movement Data Using Hidden Markov Models.” Methods in Ecology and Evolution 7, no. 11: 1308–1315. 10.1111/2041-210X.12578.

[ece370397-bib-0051] Milleret, C. , A. Ordiz , A. Sanz‐Pérez , et al. 2019. “Testing the Influence of Habitat Experienced During the Natal Phase on Habitat Selection Later in Life in Scandinavian Wolves.” Scientific Reports 9, no. 1: 1–11. 10.1038/s41598-019-42835-1. PMC6484024 31024020

[ece370397-bib-0052] Nores, C. , and J. V. López‐Bao . 2022. “Historical Data to Inform the Legal Status of Species in Europe: An Example With Wolves.” Biological Conservation 272: 109639.

[ece370397-bib-0053] Núñez‐Quirós, P. , R. García‐Lavandera , and L. Llaneza . 2007. “Análisis De La Distribución Histórica Del Lobo (*Canis lupus*) En Galícia: 1850, 1960 y 2003.” Ecología 21: 195–206.

[ece370397-bib-0054] Palacios, V. , J. V. López‐Bao , L. Llaneza , C. Fernández , and E. Font . 2016. “Decoding Group Vocalizations: The Acoustic Energy Distribution of Chorus Howls is Useful to Determine Wolf Reproduction.” PLoS One 11, no. 5: e0153858. 10.1371/journal.pone.0153858. 27144887 PMC4856277

[ece370397-bib-0055] Petroelje, T. R. , J. L. Belant , D. E. Beyer , and N. J. Svoboda . 2020. “Identification of Carnivore Kill Sites is Improved by Verified Accelerometer Data.” Animal Biotelemetry 8, no. 1: 18. 10.1186/s40317-020-00206-y.

[ece370397-bib-0056] R Core Team . 2022. *R: A Language and Environment for Statistical Computing* [Manual]. https://www.R‐project.org/.

[ece370397-bib-0057] Reichmann, A. , and D. Saltz . 2005. “The Golan Wolves: The Dynamics, Behavioral Ecology, and Management of an Endangered Pest.” Israel Journal of Zoology 51, no. 2: 87–133.

[ece370397-bib-0058] Rico, M. , and J. P. Torrente . 2000. “Caza y Rarificación Del Lobo En España: Investigación histórica y Conclusiones Biológicas.” Galemys 12: 163–179.

[ece370397-bib-0059] Rio‐Maior, H. , P. Beja , M. Nakamura , and F. Álvares . 2018. “Use of Space and Homesite Attendance by Iberian Wolves During the Breeding Season.” Mammalian Biology 92: 1–10. 10.1016/j.mambio.2018.03.014.

[ece370397-bib-0060] Saetre, P. , E. Strandberg , P.‐E. Sundgren , U. Pettersson , E. Jazin , and T. F. Bergström . 2006. “The Genetic Contribution to Canine Personality.” Genes, Brain and Behavior 5, no. 3: 240–248. 10.1111/j.1601-183X.2005.00155.x. 16594977

[ece370397-bib-0061] Salado, I. , M. Preick , N. Lupiáñez‐Corpas , et al. 2022. “Loss of Mitochondrial Genetic Diversity Despite Population Growth: The Legacy of Past Wolf Population Declines.” Genes 14, no. 1: 75. 36672816 10.3390/genes14010075 PMC9858670

[ece370397-bib-0062] Sanz‐Pérez, A. , A. Ordiz , H. Sand , et al. 2018. “No Place Like Home? A Test of the Natal Habitat‐Biased Dispersal Hypothesis in Scandinavian Wolves.” Royal Society Open Science 5, no. 12: 181379. 10.1098/rsos.181379. 30662744 PMC6304128

[ece370397-bib-0063] Sazatornil, V. , A. Rodríguez , M. Klaczek , et al. 2016. “The Role of Human‐Related Risk in Breeding Site Selection by Wolves.” Biological Conservation 201: 103–110.

[ece370397-bib-0064] SIOSE National Technical Team . 2022. High Resolution Land Cover/Land Use Information System in Spain (HR SIOSE). Dirección General del Instituto Geográfico Nacional. Subdirección General de Cartografía y Observación del Territorio. https://www.siose.es/.

[ece370397-bib-0065] Smith, B. R. , and D. T. Blumstein . 2008. “Fitness Consequences of Personality: A Meta‐Analysis.” Behavioral Ecology 19, no. 2: 448–455. 10.1093/beheco/arm144.

[ece370397-bib-0066] Theuerkauf, J. 2009. “What Drives Wolves: Fear or Hunger? Humans, Diet, Climate and Wolf Activity Patterns.” Ethology 115, no. 7: 649–657. 10.1111/j.1439-0310.2009.01653.x.

[ece370397-bib-0067] Theuerkauf, J. , W. Jȩdrzejewski , K. Schmidt , and R. Gula . 2003. “Spatiotemporal Segregation of Wolves From Humans in the Białowieża Forest (Poland).” Journal of Wildlife Management 67, no. 4: 706–716. 10.2307/3802677.

[ece370397-bib-0068] Theuerkauf, J. , W. Jȩdrzejewski , K. Schmidt , et al. 2003. “Daily Patterns and Duration of Wolf Activity in the Białowieza Forest, Poland.” Journal of Mammalogy 84, no. 1: 243–253. 10.1644/1545-1542(2003)084<0243:DPADOW>2.0.CO;2.

[ece370397-bib-0069] Tsunoda, H. , R. Gula , J. Theuerkauf , et al. 2009. “How Does Parental Role Influence the Activity and Movements of Breeding Wolves?” Journal of Ethology 27, no. 1: 185–189. 10.1007/s10164-008-0106-z.

[ece370397-bib-0070] Valverde, J. A. 1971. “El Lobo Español.” Montes 159: 229–241.

[ece370397-bib-0071] Vilà, C. 1995. “Observations on the Daily Activity Patterns in the Iberian Wolf.” In Ecology and Conservation of Wolves in a Changing World, 335–340. Edmonton, Alberta: University of Alberta Press.

[ece370397-bib-0072] Wei, T. , V. Simko , M. Levy , Y. Xie , Y. Jin , and J. Zemla . 2017. “Package ‘corrplot.’.” Stat 56, no. 316: e24.

[ece370397-bib-0073] Wickham, H. 2016. ggplot2: Elegant Graphics for Data Analysis. New York: Springer‐Verlag. https://ggplot2.tidyverse.org.

[ece370397-bib-0074] Wilson, D. S. 1998. “Adaptive Individual Differences Within Single Populations.” Philosophical Transactions of the Royal Society of London. Series B: Biological Sciences 353, no. 1366: 199–205.

[ece370397-bib-0075] Wolf, M. , G. S. van Doorn , O. Leimar , and F. J. Weissing . 2007. “Life‐History Trade‐Offs Favour the Evolution of Animal Personalities.” Nature 447, no. 7144: 581–584. 10.1038/nature05835. 17538618

[ece370397-bib-0076] Worton, B. J. 1989. “Kernel Methods for Estimating the Utilization Distribution in Home‐Range Studies.” Ecology 70, no. 1: 164–168. 10.2307/1938423.

[ece370397-bib-0077] Ylitalo, A.‐K. , J. Heikkinen , and I. Kojola . 2021. “Analysis of Central Place Foraging Behaviour of Wolves Using Hidden Markov Models.” Ethology 127, no. 2: 145–157. 10.1111/eth.13106.

[ece370397-bib-0078] Zimmermann, B. , L. Nelson , P. Wabakken , H. Sand , and O. Liberg . 2014. “Behavioral Responses of Wolves to Roads: Scale‐Dependent Ambivalence.” Behavioral Ecology 25, no. 6: 1353–1364. 10.1093/beheco/aru134. 25419085 PMC4235582

