# Supplementary material for: Drivers of Wolf Activity in a Human‐Dominated Landscape and Its Individual Variability Toward Anthropogenic Disturbance
Source: Ecol Evol. 2024 Oct 22;14(10):e70397. doi: 10.1002/ece3.70397 (PMC11494153; doi:10.1002/ece3.70397)
Supplement: Supplementary file 1 — Data S1. [file ECE3-14-e70397-s001.docx]

**Supplementary Figures**


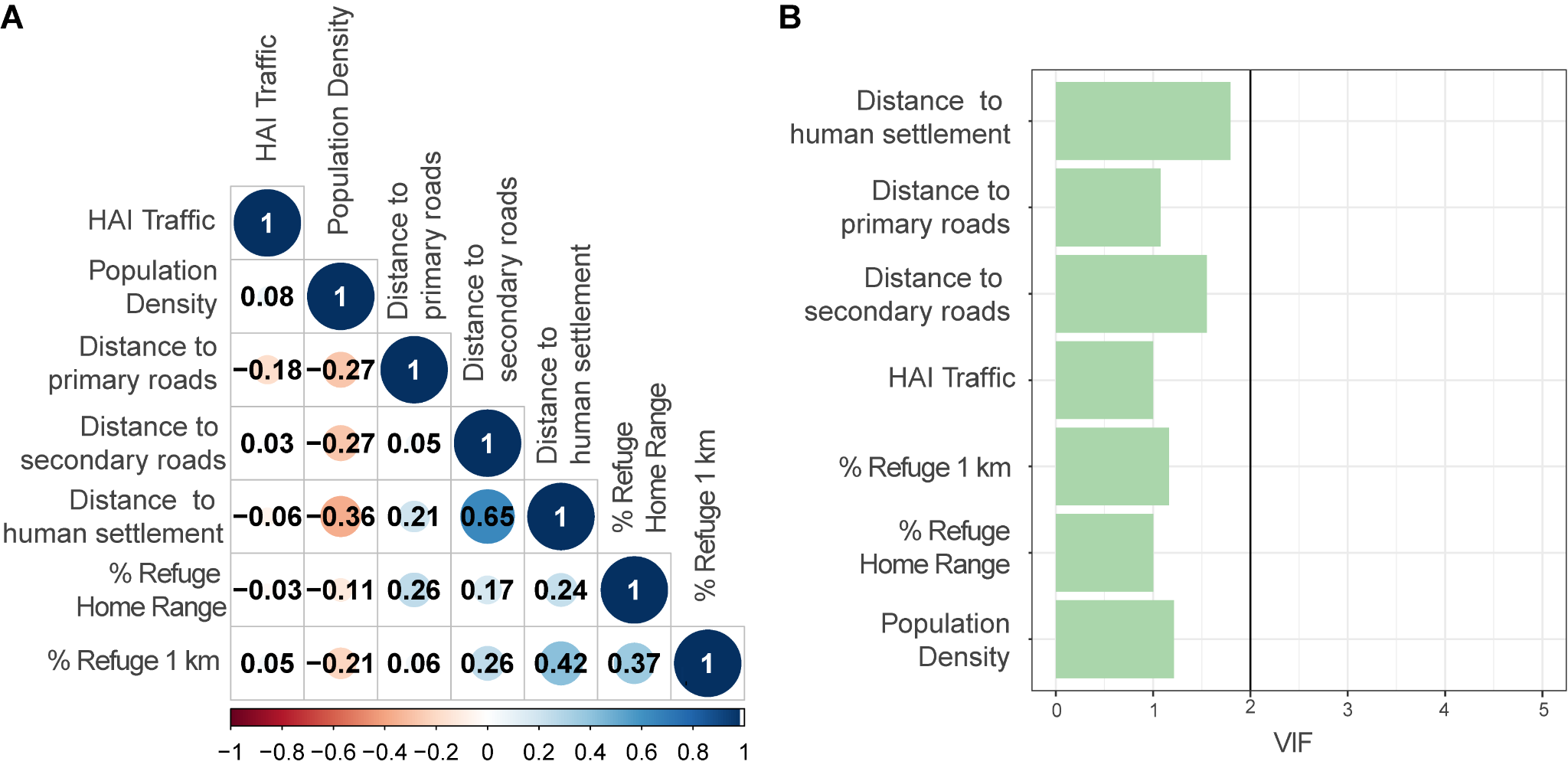


**Figure S1.** Multicollinearity assessment: pearson correlation coefficients (A) and VIF values (B).

**
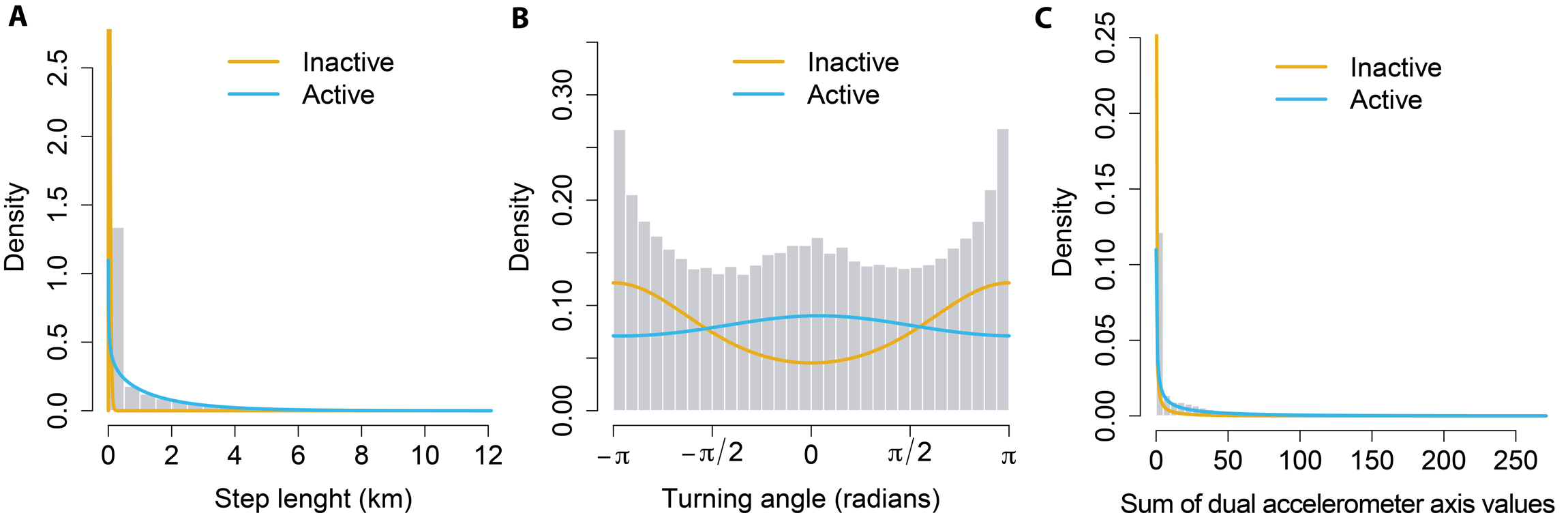
**

**Figure S2.** State-dependent distributions for “Inactive” and “Active” states derived from Hidden Markov Models expressed as function of step lengths (A), turning angles (B), and accelerometer data (C).

**Supplementary Tables.**

**Table S1.** Descriptive data of GPS sampling for each of the monitored wolves in this study to fit HMMs. Wolves L04, L21 and L43 were pack members until 15/10/2006, 01/01/2012 and 28/04/2014, respectively. From these dates, we considered them as non-pack members for analysis.

| ID | Age | Sex | Pack | Start Date | End Date | Total Days | Total track locations | Missing Locations | % Missing |
| --- | --- | --- | --- | --- | --- | --- | --- | --- | --- |
|  |  |  |  |  |  |  |  |  |  |
| L01 | Adult | Male | Yes | 24/04/2006 | 25/09/2006 | 154 | 1850 | 104 | 5.62 |
| L02 | Subadult | Male | No | 27/08/2006 | 18/10/2006 | 52 | 662 | 57 | 8.61 |
| L04 | Subadult | Male | Yes-No | 16/09/2006 | 20/12/2006 | 95 | 1145 | 175 | 15.28 |
| L05 | Subadult | Female | Yes | 27/03/2007 | 03/12/2007 | 251 | 3003 | 320 | 10.65 |
| L06 | Subadult | Male | Yes | 14/07/2007 | 17/10/2007 | 95 | 1142 | 52 | 4.55 |
| L07 | Adult | Male | No | 18/10/2007 | 26/12/2007 | 69 | 827 | 73 | 8.82 |
| L08 | Subadult | Female | Yes | 16/07/2009 | 04/11/2009 | 111 | 1333 | 121 | 90.7 |
| L09 | Adult | Male | Yes | 16/07/2009 | 25/06/2010 | 344 | 4117 | 574 | 13.94 |
| L11 | Adult | Female | Yes | 29/07/2009 | 09/06/2010 | 315 | 3683 | 99 | 2.68 |
| L12 | Subadult | Female | No | 14/08/2009 | 12/04/2010 | 241 | 2807 | 183 | 6.52 |
| L13 | Subadult | Male | Yes | 19/11/2009 | 11/03/2010 | 112 | 1360 | 115 | 8.45 |
| L17 | Subadult | Female | Yes | 11/04/2011 | 01/09/2011 | 143 | 1719 | 155 | 9.02 |
| L18 | Subadult | Female | Yes | 11/04/2011 | 27/08/2011 | 138 | 1636 | 213 | 13.01 |
| L19 | Subadult | Female | Yes | 05/04/2011 | 22/08/2011 | 139 | 1671 | 255 | 15.26 |
| L20 | Adult | Female | Yes | 28/06/2011 | 27/10/2011 | 60 | 1450 | 169 | 11.65 |
| L21 | Adult | Male | Yes-No | 10/07/2011 | 04/04/2012 | 269 | 3237 | 46 | 1.42 |
| L22 | Subadult | Female | Yes | 14/07/2011 | 14/08/2012 | 397 | 4769 | 40 | 0.84 |
| L23 | Subadult | Female | Yes | 13/08/2011 | 06/01/2012 | 146 | 1751 | 11 | 0.63 |
| L24 | Adult | Female | Yes | 17/08/2011 | 16/11/2011 | 91 | 1098 | 46 | 4.19 |
| L27 | Adult | Male | No | 27/12/2011 | 07/06/2012 | 178 | 1950 | 72 | 3.69 |
| L28 | Adult | Male | Yes | 16/07/2012 | 02/04/2013 | 259 | 3119 | 264 | 8.46 |
| L36 | Subadult | Male | Yes | 10/07/2013 | 13/05/2014 | 307 | 3688 | 909 | 24.64 |
| L37 | Subadult | Male | Yes | 09/08/2013 | 16/06/2014 | 327 | 3733 | 599 | 16.04 |
| L38 | Subadult | Male | Yes | 15/08/2013 | 24/01/2014 | 162 | 1943 | 319 | 16.42 |
| L41 | Adult | Female | Yes | 12/03/2014 | 22/12/2014 | 285 | 3423 | 450 | 13.15 |
| L43 | Subadult | Male | Yes-No | 20/03/2014 | 31/12/2014 | 286 | 3423 | 397 | 11.60 |

**Table S2.** Hypothesis and expected relationships between wolf activity and proxies of anthropogenic disturbance.

| **Predictor** | **Hypothesis** | **Sources** |
| --- | --- | --- |
| Interaction between Human Population Density and day period | Wolf activity patterns are influenced by human population density, leading to temporal adjustments in their behavior to avoid human encounters. Wolves are known to adjust their behavior in response to human presence to reduce the risk of conflict. High human population densities increase the likelihood of encounters between wolves and humans during the day. In contrast, in sparsely populated areas, the lower risk of encountering humans would allow wolves to be more active during daylight hours. Hence, we hypothesize that on areas with high human population densities, wolves will demonstrate a significant reduction in their daylight activity, opting for increased nocturnal behavior to minimize the risk of encountering humans. Conversely, in areas with low human population densities, wolves will exhibit higher levels of daylight activity, as the risk of encountering humans is reduced. | ^1–4^ |
| Interaction between Euclidean distance to human settlements and day period | Wolves adapt their behaviour to mitigate the risk of encountering humans. The proximity to human settlements presents a higher risk of human-wolf interactions, particularly during the day when human activity is at its peak. Therefore, wolves are expected to avoid these areas during daylight hours. At night, when human activity diminishes, wolves may take advantage of the reduced risk to explore areas closer to human settlements in search of food or other resources. Specifically, we hypothesize that during the daytime, wolves will avoid areas closer to human settlements, leading to a decreased probability of wolf activity in these sites. Conversely, at night, wolves are more likely to approach human settlements, as decreased human activity reduces the risk of encounters. | ^5,6^ |
| Interaction between Euclidean distance to primary roads and day period | Wolves’ responses to paved roads were reported to vary among day periods, taking advantage of crossing roads when traffic volume is lower (i.e. night periods). We expect that wolves will modulate their activity in response to the proximity of paved roads and the time of day, taking advantage of lower traffic volumes during nighttime for road crossings. Primary roads, with their high traffic volumes during the day, pose significant risks to wolves, potentially leading to a reduction in diurnal activity near these areas when the traffic intensity is at its peak. At night, the decrease in traffic would allow wolves to approach and cross roads more safely, thus increasing their nocturnal activity near roads. Specifically, we hypothesize that during daylight hours, wolves will reduce their activity in areas close to primary roads due to elevated traffic volumes and human activity, resulting in a lower probability of wolf activity near roads. As the distance from primary roads increases, diurnal wolf activity is expected to rise, reflecting reduced human disturbance. Conversely, during nighttime, wolves are expected to increase their activity near primary roads, taking advantage of reduced traffic and human presence to cross roads or move through these areas. At greater distances from primary roads, nocturnal wolf activity is anticipated to decrease, as the need to avoid high-traffic areas diminishes. | ^5–7^ |
| Interaction between Euclidean distance to secondary roads and day period | Wolves are known to exhibit adaptive behaviour to avoid human encounters, which is influenced by the level of human activity associated with different types of roads. While secondary roads have lower traffic volumes and human presence compared to primary roads, wolves still adjust their spatial and temporal activity to minimize risk. We expect that, during the day, when human activity is higher, wolves are likely to avoid areas near secondary roads, although this avoidance is less pronounced than with primary roads. At night, the reduction in human activity allows wolves to utilize areas closer to secondary roads more freely, reflecting an opportunistic strategy to access resources while minimizing risks. Specifically, we hypothesize that, during daylight hours, wolves will demonstrate a positive relationship between activity and distance from secondary roads, with increased diurnal activity as the distance from these roads increase. This would reflect a tendency to avoid areas with higher human activity during the day, even though secondary roads have lower traffic volumes compared to primary roads. Conversely, during nighttime, wolves will increase their activity closer to secondary roads, taking advantage of the lower human presence and reduced traffic during these periods. This results in a higher probability of nocturnal activity near secondary roads. | ^5–7^ |
| Hourly Average Traffic Volume | Wolves are known to adjust their activity patterns in response to human presence to minimize the risk of encounters and such adjustments may be scale-dependent. We employed the Hourly Average Traffic Volume, as a proxy for human activity at the home-range scale which serves as a dynamic measure of human activity, reported for different road segments every hour of each day of the week. This measure allows for precise matching of human activity levels and its temporal patterns at home-range scale with records of wolf activity, providing a detailed picture of how wolves adapt their behavior. We expect that the elevated traffic volumes during certain hours indicate higher human presence and activity within the wolves' home ranges. During these periods, the likelihood of human-wolf interactions increases, prompting wolves to reduce their activity to avoid potential conflicts. Consequently, we further hypothesize that wolves will shift their activity to nighttime hours when traffic volumes and human activity are significantly higher, thus reducing the risk of encounters and taking advantage of safer conditions for movement and foraging. | ^6,7^ |
| Refuge cover within 1km | Wolves' responses to refuge cover can be scale-dependent and contextually complex. At a fine scale (within 1 km), vegetation cover provides critical refuge, enabling wolves to be more active during daylight hours by reducing the risk of encounters and disturbance. However, they also select sites with ample vegetation cover as a resting site. This dual role of vegetation cover—both as a refuge and a resting area—can complicate the direct relationship between cover availability and activity levels. Specifically, we hypothesize that when ample vegetation cover is available within 1 km, the probability of wolf activity during daylight will increase due to the refuge provided by the vegetation. However, this increase in activity may be moderated by the wolves' use of such cover for resting and refuge, potentially leading to a reduction in activity. In areas with abundant cover, wolves may balance their diurnal activity with increased use of the cover for resting, which can mask the positive effect of refuge cover on activity levels and diminish the differences in activity between day and night periods. Conversely, when vegetation cover within 1 km is scarce, wolves will likely reduce their diurnal activity and increase nocturnal activity to avoid higher risks associated with limited cover and increased human presence during the day. | ^8,9^ |
| Interaction between Human Population Density and Refuge Cover at home-range scale | Refuge cover within a wolf’s home range provides critical protection from human disturbance, offering a safe haven for wolves to engage in activity. Yet, wolf responses to refuge availability may be scale-dependent. Effects of fine-scale refuge cover might be confounded with habitat-selection processes, where wolves might choose areas with better cover but not necessarily reflect overall buffering capacity. In contrast, refuge cover at the home-range scale captures the broader context in which wolves operate, offering a more accurate representation of how vegetation cover buffers the effects of human presence. Hence, the impact of human population density on wolf activity will be moderated by the amount of refuge cover available within the home range. Specifically, we hypothesize that an ample refuge cover at the home-range scale will buffer or mitigate the negative effects of high human population density on overall wolf activity. However, we expect that in areas with limited refuge cover, the negative impact of high human population density on wolf activity may be magnified, resulting in more pronounced reductions in overall activity. | ^6,8,9^ |

**Supplementary References**

1. Theuerkauf, J. *et al.* Daily Patterns and Duration of Wolf Activity in the Białowieza Forest, Poland. *Journal of Mammalogy* **84**, 243–253 (2003).

2. Martínez-Abraín, A. *et al.* Increased grey wolf diurnality in southern Europe under human-restricted conditions. *Journal of Mammalogy* **104**, 846–854 (2023).

3. Theuerkauf, J. *et al.* Human impact on wolf activity in the Bieszczady Mountains, SE Poland. *Annales Zoologici Fennici* **44**, 225–231 (2007).

4. Ciucci, P., Boitani, L., Francisci, F. & Andreoli, G. Home range, activity and movements of a wolf pack in central Italy. *Journal of Zoology* **243**, 803–819 (1997).

5. Mancinelli, S., Falco, M., Boitani, L. & Ciucci, P. Social, behavioural and temporal components of wolf (Canis lupus) responses to anthropogenic landscape features in the central Apennines, Italy. *Journal of Zoology* **309**, 114–124 (2019).

6. Theuerkauf, J., Jȩdrzejewski, W., Schmidt, K. & Gula, R. Spatiotemporal Segregation of Wolves from Humans in the Białowieża Forest (Poland). *The Journal of Wildlife Management* **67**, 706–716 (2003).

7. Dennehy, E., Llaneza, L. & López-Bao, J. V. Contrasting wolf responses to different paved roads and traffic volume levels. *Biodiversity and Conservation* **30**, 3133–3150 (2021).

8. Grilo, C. *et al.* Refuge as major habitat driver for wolf presence in human-modified landscapes. *Animal Conservation* **22**, 59–71 (2019).

9. Llaneza, L., García, E. J., Palacios, V., Sazatornil, V. & López-Bao, J. V. Resting in risky environments: the importance of cover for wolves to cope with exposure risk in human-dominated landscapes. *Biodiversity and Conservation* **25**, 1515–1528 (2016).
